# Case Report: Human metapneumovirus infection leading to septic shock in an adult with COPD and morbid obesity

**DOI:** 10.3389/fmed.2025.1735031

**Published:** 2025-12-18

**Authors:** Francesco Virzì, Giuseppe Giacopelli, Paolo Tutone, Cecilia Riccobono, Luigi Profera, Santi Maurizio Raineri, Sandro Tomasello, Giuseppe Accurso

**Affiliations:** 1 Unit of Anesthesia, Intensive Care, and Hyperbaric Medicine, Civico Emergency Hospital, Partinico, Sicily, Italy; 2 Department of Anaesthesia, Intensive Care and Emergency, Policlinico Paolo Giaccone, Palermo, Sicily, Italy; 3 Department of Precision Medicine in Medical, Surgical and Critical Care, University of Palermo, Palermo, Sicily, Italy

**Keywords:** human metapneumovirus, viral pneumonia, COPD, obesity, septic shock, case report

## Abstract

**Background:**

Human metapneumovirus (hMPV) is an emerging respiratory pathogen causing illnesses from mild upper respiratory infection to severe pneumonia, particularly in high-risk adults. Although primarily recognized in pediatrics, hMPV is increasingly reported as a cause of acute respiratory failure in adults with chronic lung disease and obesity.

**Case:**

A 58-year-old man with morbid obesity and chronic obstructive pulmonary disease presented with acute respiratory failure and viral septic shock. Molecular testing of bronchoalveolar lavage (BioFire® FilmArray®) identified *human metapneumovirus* type 1 as the sole pathogen. The patient required invasive mechanical ventilation, vasopressor support, and continuous renal replacement therapy. Vancomycin was discontinued for suspected nephrotoxicity, and systemic corticosteroids were introduced. Subsequent bronchoalveolar lavage cultures grew *Streptococcus pneumoniae*, confirming bacterial superinfection. With supportive management, the patient gradually improved, was extubated on day 9, and was discharged home in stable condition.

**Conclusion:**

hMPV can trigger severe respiratory failure and multiorgan dysfunction in adults with chronic respiratory comorbidities. Early molecular diagnosis is crucial to guide management and limit unnecessary antimicrobial exposure. The absence of specific antivirals underscores the need for continued research into targeted treatments and preventive vaccines.

## Introduction

Human metapneumovirus (hMPV) is an emerging respiratory pathogen that has gained increasing clinical relevance in adult intensive care. While most infections are self-limiting, hMPV can occasionally cause severe respiratory failure and viral sepsis in adults with chronic lung disease or obesity. This report describes a critically ill patient who developed hMPV-related septic shock complicated by bacterial superinfection. To our knowledge, this is one of the first detailed reports of hMPV-1–associated viral sepsis in an adult with morbid obesity following the 2025 WHO alert.

## Case presentation

A 58-year-old male was admitted to the Emergency Department of the Civico Hospital of Partinico (Italy) in February 2025 with acute severe respiratory distress. His medical history included morbid obesity (BMI > 45), chronic obstructive pulmonary disease (COPD), bronchiectasis, chronic respiratory failure on home non-invasive ventilation and nocturnal oxygen therapy, ischemic heart disease secondary to long-standing hypertension, type 2 diabetes mellitus, and chronic venous insufficiency. His home medications comprised a sodium–glucose cotransporter-2 inhibitor, a GLP-1 receptor agonist, low-dose aspirin, a statin, omega-3 fatty acids, a proton-pump inhibitor, an ACE inhibitor, a venotonic agent, long-acting insulin, and an antihyperuricemic drug. He was regularly vaccinated against *Streptococcus pneumoniae*, SARS-CoV-2 (four doses), and seasonal influenza.

On arrival, the patient appeared critically ill, with severe acute respiratory failure and rapidly progressive cognitive decline (Glasgow Coma Scale 9). Physical examination revealed peripheral cyanosis, diaphoresis, tachypnea with paradoxical breathing and use of accessory muscles, hypertension (160/100 mmHg), tachycardia (112 bpm), and anuria. Lung auscultation demonstrated diffuse wheezes and reduced breath sounds at the bases, consistent with severe obstructive pneumopathy overlapping with restrictive mechanics due to obesity. Diffuse folliculitis was also observed.

Arterial blood gas analysis showed severe respiratory acidosis with marked hypercapnia. Laboratory tests revealed leukocytosis, elevated C-reactive protein, and normal procalcitonin levels. Because of worsening dyspnea and declining mental status, the patient was sedated, intubated, and mechanically ventilated. Lung-protective ventilation was instituted according to ARDSNet recommendations (1), but gas exchange remained severely impaired, consistent with refractory hypoxemic and hypercapnic respiratory failure. The patient was therefore admitted to the intensive care unit (ICU) for ongoing management.

**Figure 1:**
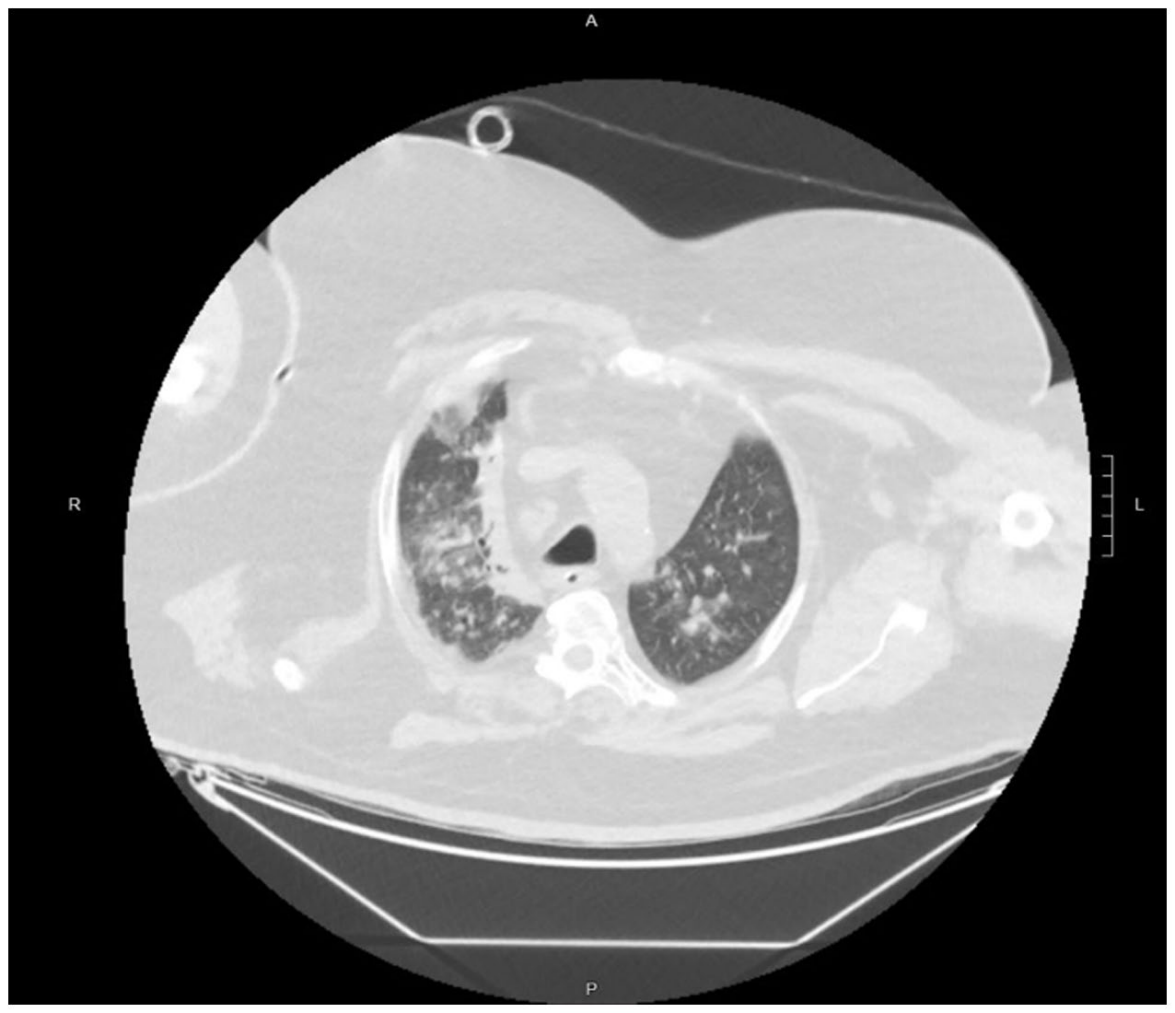
Chest computed tomography (CT) on ICU admission. Extensive confluent parenchymal consolidations with air bronchograms are visible in the right lung, associated with diffuse ground-glass opacities and “tree-in-bud” patterns in the left upper lobe—findings consistent with bilateral viral pneumonia.

Microbiological samples, including bronchoalveolar lavage (BAL) and blood cultures, were promptly collected. Empiric broad-spectrum antibiotics (piperacillin–tazobactam and vancomycin) were initiated according to ICU protocols. A total-body computed tomography (CT) scan revealed extensive confluent parenchymal consolidations in the right lung with air bronchograms and “tree-in-bud” opacities in the left upper lobe—findings compatible with bilateral viral pneumonia (Figure 1).

A few hours after ICU admission, results from the syndromic multiplex PCR assay (BioFire® FilmArray®) performed on the BAL became available, revealing exclusive positivity for *human metapneumovirus* type 1. Over the following 48 h, the patient’s condition deteriorated further. He developed viral septic shock with profound hemodynamic instability requiring continuous vasopressor support. Persistent anuria and rising azotemia prompted the initiation of continuous renal replacement therapy.

Vancomycin was discontinued due to suspected nephrotoxicity, and antimicrobial coverage was empirically adjusted to imipenem/cilastatin to ensure broad Gram-negative and Gram-positive coverage during ongoing diagnostic reassessment. Systemic corticosteroids were initiated due to a rapidly worsening inflammatory profile, severe viral-associated ARDS physiology, and progressive hemodynamic deterioration. Fluconazole was preferred over echinocandins because of its favorable renal safety profile during CRRT.

Approximately 72 h after admission, the patient’s respiratory and hemodynamic parameters began to improve. BAL cultures subsequently grew *Streptococcus pneumoniae*, not detected by the initial multiplex PCR, confirming bacterial superinfection. Serologic pneumococcal vaccine seroconversion and pneumococcal strain typing were not performed, as these analyses are not routinely available in our institution. The multiplex respiratory PCR panel was negative for all other viruses and atypical bacteria, and blood cultures remained negative, supporting hMPV-1 infection as the primary cause of respiratory failure.

**Figure 2:**
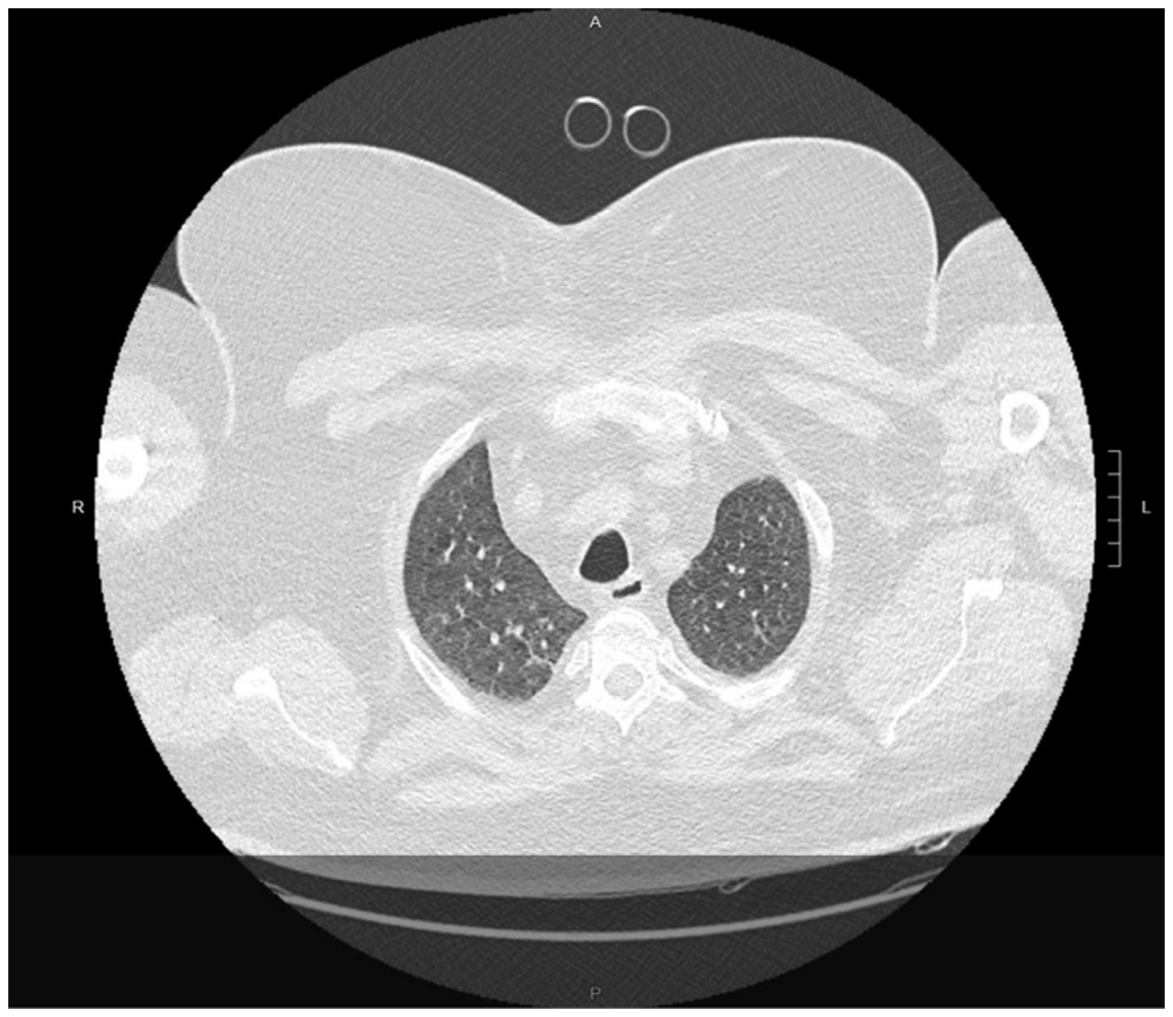
Follow-up chest computed tomography after clinical improvement. Marked resolution of the parenchymal consolidations and ground-glass opacities is observed following supportive and antimicrobial therapy, consistent with recovery from hMPV-related pneumonia.

Antimicrobial therapy was maintained according to susceptibility results until full recovery. The patient’s clinical condition continued to improve over the following days, allowing discontinuation of renal replacement therapy and, on day 9, successful extubation after a spontaneous breathing trial (Figure 2). He was transferred to a medical ward and later discharged home in stable condition after full respiratory recovery. A summary of the patient’s clinical course, major interventions, and outcomes is presented in Table 1.

**Table 1:** Timeline of clinical course, laboratory trends, and major interventions.

Day	Event	Key findings
0	ED presentation	pH 7.0; PaCO₂ 124 mmHg; P/F ≈ 124; WBC 12,900/mm^³^ (82% neutrophils); CRP 11 mg/dL; PCT < 0.3 ng/mL; Na^+^ 150 mEq/L; anuria
0–1	ICU admission	Mechanical ventilation; empiric antibiotics; persistent hypoxemia/hypercapnia
1–2	Clinical deterioration	Temp 39 °C; MAP ≈35 mmHg; norepinephrine ↑ to 1 μg/kg/min; creatinine 1.4 → 2.9 mg/dL; hyperkalemia; CRP ↑ to ~18 mg/dL; P/F < 60
2	CRRT + steroids	Prednisone 25 mg/day; CRRT started; vancomycin stopped
3 (72 h)	Initial improvement	pH 7.16; PaCO₂ 80; PaO₂ 78; P/F 87; WBC 13,000/mm^³^; CRP 8.2 mg/dL; PCT 0.9
4 (96 h)	Further improvement	pH 7.3; PaCO₂ 50; PaO₂ 80; SpO₂ 95.4% (FiO₂ 70%); lactate 0.8; afebrile
5	Renal stabilization	CRRT discontinued; anuria persists
9	Extubation	Successful SBT; stable ABG; afebrile
14	Discharge	Full recovery

## Discussion

Human metapneumovirus (hMPV) has emerged as a relevant cause of severe lower respiratory tract infections not only in children but also in adults with chronic comorbidities. Recent evidence shows that hMPV accounts for a significant proportion of hospitalizations for acute respiratory illnesses in adults, particularly in those with COPD, heart failure, or obesity (2). The present case illustrates how hMPV infection can precipitate acute respiratory failure and multiorgan dysfunction in a patient with COPD and morbid obesity, both recognized risk factors for severe disease (3, 4).

From a pathophysiological perspective, the severe course observed in such patients can be explained by the combined effects of direct viral cytopathic damage and an exaggerated host inflammatory response. hMPV primarily infects respiratory epithelial cells, causing ciliary dysfunction, epithelial detachment, and syncytia formation. These alterations impair mucociliary clearance and facilitate bacterial adhesion and secondary infection. Moreover, COPD and obesity are associated with chronic airway inflammation and impaired interferon-mediated immunity, which may amplify cytokine production (notably IL-6, TNF-α, and IL-8), worsening pulmonary injury and contributing to acute respiratory distress. As highlighted in recent reviews, this dysregulated immune activation represents a hallmark of severe hMPV disease (2). A similar interplay between respiratory mechanics, inflammatory burden, and clinical deterioration has been described in previous studies exploring ventilatory monitoring strategies in acute respiratory failure (5).

In our case, the subsequent isolation of *Streptococcus pneumoniae* from bronchoalveolar lavage supports the hypothesis that hMPV can act as a “viral gateway” for bacterial superinfection—a mechanism well documented in both pediatric and adult populations. Such viral–bacterial synergy is increasingly recognized as a determinant of clinical deterioration and septic shock in viral pneumonia (2). Pneumococcal serotype analysis was not available; therefore, we could not determine whether infection occurred despite vaccine serotype coverage.

Currently, there are no licensed antiviral agents or vaccines for hMPV. Management remains largely supportive, consisting of oxygen supplementation, mechanical ventilation when necessary, and optimization of comorbidities. Experimental therapeutic options include broad-spectrum antivirals such as ribavirin and remdesivir, small-molecule polymerase inhibitors, and monoclonal antibodies targeting the F protein, which have shown cross-neutralizing activity against both hMPV and RSV in preclinical studies. In addition, multivalent vaccine candidates targeting the F protein of hMPV and RSV are under development, with encouraging early results (9–11). Corticosteroid therapy, although not specific for hMPV, may help modulate the inflammatory response and has been associated with clinical improvement in selected severe cases (1, 2, 4, 6–7).

This case—the first documented hMPV-1 infection reported within the Palermo province following the WHO Disease Outbreak News (January 2025) (8)—reinforces the need to include hMPV in the differential diagnosis of severe respiratory infections in adults. Early molecular diagnosis through multiplex PCR enables rapid identification, prevents unnecessary antibiotic escalation, and guides supportive management. Heightened clinical awareness and strengthened surveillance networks are essential to improve outcomes and to promote the development of specific antiviral and preventive strategies against hMPV (2).

To contextualize this case, we performed a targeted literature search using PubMed and Google Scholar (keywords: “human metapneumovirus AND adult,” “hMPV AND ICU,” “viral sepsis,” “hMPV AND superinfection”), covering the years 2001–2025.

## Conclusion

This case highlights the importance of recognizing the synergistic interaction between hMPV and bacterial superinfection, which can rapidly precipitate respiratory failure and multiorgan dysfunction.

### Limitations

This report describes a single patient and lacks pneumococcal serotype testing and viral-load quantification. Findings may not be generalizable to all adults with hMPV, particularly those without chronic respiratory disease.

### Patient perspective

The patient expressed gratitude for the medical care received and reported full respiratory recovery without any long-term complications.

## Data Availability

The original contributions presented in the study are included in the article/Supplementary material, further inquiries can be directed to the corresponding author.
